# Protecting the peri-domestic environment: the challenge for eliminating residual malaria

**DOI:** 10.1038/s41598-020-63994-6

**Published:** 2020-04-27

**Authors:** Edgar J. M. Pollard, David MacLaren, Tanya L. Russell, Thomas R. Burkot

**Affiliations:** James Cook University, Australian Institute of Tropical Health and Medicine, Cairns, QLD 4870 Australia

**Keywords:** Ecological epidemiology, Parasitology, Malaria

## Abstract

Malaria transmission after universal access and use of malaria preventive services is known as residual malaria transmission. The concurrent spatial-temporal distributions of people and biting mosquitoes in malaria endemic villages determines where and when residual malaria transmission occurs. Understanding human and vector population behaviors and movements is a critical first step to prevent mosquito bites to eliminate residual malaria transmission. This study identified where people in the Solomon Islands are over 24-hour periods. Participants (59%) were predominantly around the house but not in their house when most biting by *Anopheles farauti*, the dominant malaria vector, occurs. While 84% of people slept under a long-lasting insecticide-treated bed net (LLIN), on average only 7% were under an LLIN during the 18:00 to 21:00 h peak mosquito biting period. On average, 34% of participants spend at least one night away from their homes each fortnight. Despite high LLIN use while sleeping, most human biting by *An. farauti* occurs early in the evening before people go to sleep when people are in peri-domestic areas (predominantly on verandas or in kitchen areas). Novel vector control tools that protect individuals from mosquito bites between sundown and when people sleep are needed for peri-domestic areas.

## Introduction

Transmission of mosquito-borne diseases depends on human–vector contact^1,2^. The concurrent movements and activities of both the human and vector populations that bring these two populations in contact will define the intensity of malaria transmission^1,3–6^. After universal access and use of malaria preventive servicesis achieved, eliminating residual malaria transmission requires appropriate interventions to disrupt the remaining human-vector contact^4,7–12^.

Malaria transmission in a specific location is determined by the lifestyle and movements of the residents (among houses, workplaces, neighbourhoods, villages, towns), coupled with mosquito biting patterns through space^13^ and time^14^. People move in and around the specific local areas where they live and work and also beyond their local environs. When people move beyond the immediate range of a mosquito’s flight, wider dispersals or acquisitions of malaria and other human vector-borne pathogens can occur^5,15,16^. Investigating how people move on a broad scale, which can be periodic and/or seasonal across regional, intra-national or international scales^6,9,17^ helps to understand broad scale malaria transmission and risk. Investigating how people move within more specific local areas such as in/around households, villages or neighbourhoods is critical to understanding and preventing local malaria transmission in specific locations. This understanding of local level transmission is of particular importance when striving to eliminate residual malaria transmission.

As overall malaria transmission is reduced at a provincial, national or regional level, residual transmission becomes highly heterogeneous and local transmission foci emerge^18,19^. In the South Pacific nation of the Solomon Islands^13^, malaria transmission varies at two scales, an inter- and at an intra-village scale. Within villages, mosquito biting is highly heterogeneous and understanding the locations where residents spend the majority of their time during periods of peak mosquito activity will define their local risk of malaria. In particular, the amount of time that people spend inside households directly relates to protection from biting mosquitoes and malaria in the Solomon Islands. This protective house effect is a function of two factors. Firstly, the two WHO recommended malaria vector control tools, long lasting insecticidal nets (LLINs) and indoor residual spraying (IRS), only provide protection to individuals inside houses, either when sleeping under an LLIN or inside a room that was sprayed with insecticides (e.g., IRS). The second factor relates to the biting behaviour of the malaria vector, *Anopheles farauti*. *Anopheles farauti* is temporally and spatially heterogeneous^13^ and bites predominantly outdoors and early in the evening^14^. Thus, individuals are far less likely to be bitten when they are inside a house, even if the house is without screening or other mosquito exclusion methods. People nearby but outside the house, in the peri-domestic area, do not receive the protective benefits of LLIN/IRS and are exposed to higher biting rates by *An. farauti* due to the mosquito’s preference for biting outdoors.

At the Solomon Islands provincial level, two provinces (Isabel and Temotu) are nearing malaria elimination while other provinces continue to have high rates of transmission. People moving out of Isabel and Temotu provinces are at increased risk of malaria infection. Movement of infected individuals into these provinces threatens  elimination efforts by introducing malaria parasites. To date, only one study has explored human movement in the Solomon Islands. This study focused on Isabel Province, and documented broad-scale travel between villages/towns within and beyond the province^20^. However, fine scale movement of people within villages remains undocumented.

To inform a better understanding of how people move within specific residual transmission foci in the Solomon Islands, this study documented how people moved at both the specific local scale (in/around households within villages) during the period from 18.00 to 00.00 h when 93% of *An. farauti* bites occur^21^ and at a broader scale (beyond the village) using individual movement diaries. Documenting where people are in the evening and night (18.00-0.00 h) helps understand exposure to malaria vectors in the village. However, knowledge of people movement across a 24-hour period helps understand broader social and environmental movements as well as exposure to other vectors such as the *Aedes* vectors of dengue, Zika and chikungunya viruses which are active during the day. Data on people locations over a 14-day period in two villages were compiled, with the analyses in this study focusing on quantifying human-vector interactions^10^ where and when humans are exposed to the bites of malaria vectors.

## Results

Human movement data was collected from a total of 1204 person-days. Participants were children <5 years (11%), youth 5 to 18 years (37%) and adults >18 years (52%). The age structure of survey population was representative of the national population, and there was no difference in the age structure between either population (χ^2^ = 1.276, df = 2, p = 0.528). All households had LLINs with an average of almost 4 LLINs per household. There were 1.4 persons/LLIN. Although 84% of people reported sleeping under an LLIN, only 7% could be under an LLIN during the 18:00 to 21:00 h peak mosquito biting period. Window screens were present in only one house.

### Overall people movement

**Figure 1:**
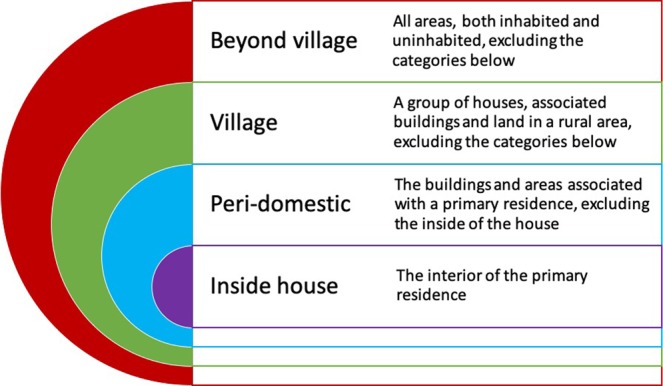
Schematic detailing the set of nested categories where people were located.

**Figure 2:**
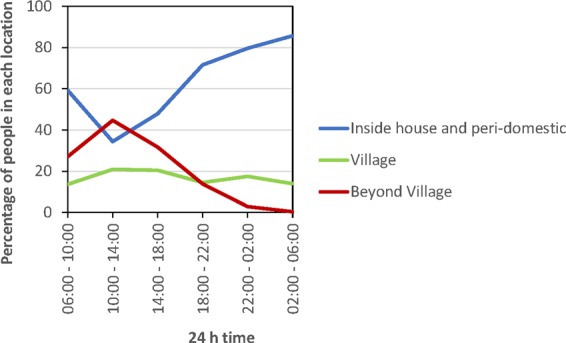
The 24 h profile of human movement within the three categories of at home (includes both inside house and peri-domestic), within village and beyond village.

People locations were designated as being within one of four nested categories, of increasing scale: inside the house, the peri-domestic area around the house (including the veranda and external kitchen building), the residential village and all areas beyond the village of residence (Fig. 1). Each nested location category was composed of finer scale sub-locations (Table 1). There were significant changes in the location of people during a 24 hr period with most people in or near their homes in the early morning as well as in the late afternoon and at night; however, in the middle of the day more people were outside their village or within their village but away from the house and peri-domestic area and these changes in locations over 24-hour periods were statistically different (χ^2^ = 768.17, df = 6, p = <0.0001; Fig. 2).

**Table 1:** Categorisation of locations where people were located.

Broad locations	Sub-locations	Descriptions
Inside house	*The interior of the building (often elevated on posts) used by a family as the primary residence with four walls enclosing one or more rooms under a roof, and may have, as follows:*	
	Bed room	An area of a house defined by four walls that is used primarily for sleeping.
	Living room	An area of a house defined by four walls that is used for purposes other than sleeping.
Peri-domestic	*A cluster of outbuildings and the associated area of land used by the residing family. Here, the peri-domestic is associated with a primary residence (is external to the nested category inside house) and includes the following:*	
	Kitchen	A roofed structure that may have walls, separated from the house and used primarily for cooking and dining.
	Outside areas	The land adjacent to the house not covered by a roof.
	Under house	An open sheltered area beneath the floor of the house.
	Veranda	A sheltered platform along the outside of the house, level with house flooring.
Village	*The smallest administrative unit in a rural area encompassing a cluster of residential houses(but external to the nested category peri-domestic) and other buildings including:*	
	Another house	Any house that is not the residence of the participant and is situated within the confines of the village.
	Church	The building and adjacent area of land where religious activities are held.
	Freshwater	Areas where water is collected for consumption or washing (e.g., well, water tank).
	School	The building and adjacent area of land where educational activities take place.
	Seaside	The coastal areas bordering the ocean.
	Store	A building that sells a variety of food and household items.
Beyond Village	*All geographic areas beyond the village which may be proximal (and frequented by day trips) or distal to the village (whose visitation often necessitates being away overnight from the home village) and includes the following:*	
	Another village	Clusters of rural houses away from the home village.
	Forest	Uncultivated or forested areas where hunting and gathering occurs.
	Garden/Farm/ Plantation	A cultivated area of land, often near the village, where food is grown.
	Town or City	Urban areas characterised with larger populations, denser housing, commercial activities and government services.
	Sea	Marine area where saltwater fish are caught.

### Overnight people movement

The overnight period was analysed to document if people spent the night within their home village or outside their village. During the 14-day study, 34% (n = 29) of participants spent at least one night away from the village. Almost equal numbers of males (n = 15) and females (n = 14) spent at least one night away from their village. The average number of nights away from the village was 3.6 per fortnight (range = 1 to 12) with no significant differences by gender (females μ = 3.0 and males μ = 4.1 nights away; β = 1.066, se = 1.245, p = 0.399) or age (compared with the baseline age distribution of all participants) (β = 1.122, se = 3.601, p = 0.756). The average number of nights that participants spent away from the village differed by village (Haleta village μ = 1.2 and Tuguivili village μ = 4.6 nights away; β = 3.378, se = 1.197, p = 0.008). The most frequent overnight travel location was to another village (59%) followed by a town or city (24%) and for employment on ships (14%).

### Night-time people movement (18:00–06:00 h)

**Figure 3:**
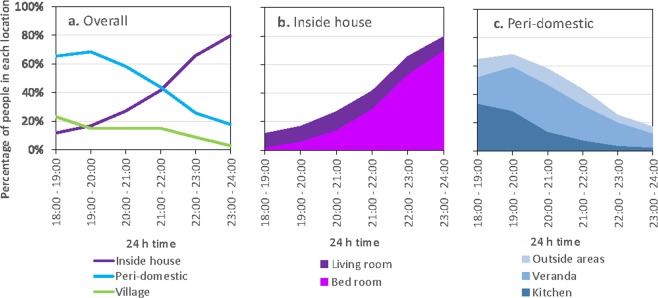
The profiles of human movement across the evening for: (**a**) within the three nested categories of inside the house, the peri-domestic area and the village; (**b**) the sub-categories for inside the house; and (**c**) the sub-categories for the peri-domestic environment. Note that the stacked profiles for graphs b and c are calculated as a breakdown of the percentages presented for each category in graph a.

The night-time period (18:00–06:00 h) was analysed in 1-hour blocks for the evening time period between 18:00–00:00 h and then a single 6-hour late night-time block between 00:00–06:00 h. Locations at specific times were recorded in 3 categories: the house, the peri-domestic area and the village (people who were beyond the village for the evening were not included in the ‘internal village’ movement analysis). There were significant differences in the locations of participants during the evening hours between the three location categories (18:00–00:00 h) (χ^2^ = 1762.3, df = 10, p = <0.0001), across all days and ages. At 18:00 h 12% were inside the house, 65% were in the peri-domestic area and 23% were in the village (Fig. 3a). During 18:00–21:00 h, half (53%) of participants were in two peri-domestic locations, the kitchen (25%) and the veranda (28%) (Fig. 3c). During 21:00–00:00 h, 62% of people were inside the house (with almost 80% in a bedroom) (Fig. 3b), 29% in the peri-domestic area and 9% in the village. At 00:00 h 80% were inside the house, 17% were in the peri-domestic area, 3% were in the village. Between 00:00 h and 06:00 h, 94% were inside the house, 5% were in the peri-domestic area, 1% were in the village. There was no statistical difference between weekends and weekdays for the percentage of people that were inside the house over the evening (eg. people inside house at 20:00 − 21:00: weekdays = 23% weekends 25%).

### Differences between age groups

**Figure 4:**
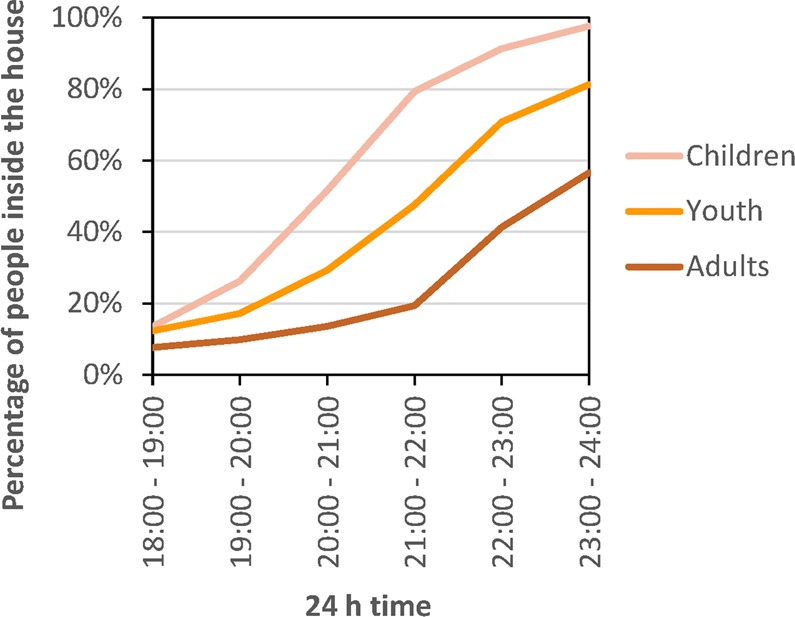
The evening profile of participants located inside the house for each age category.

The number and proportion of participants inside the house across the evening varied by age (χ^2^ = 39.526, df = 10, p < 0.0001). Ultimately, 100% of under 5 year olds, 99% of 6–18 year olds and 89% of adults (χ^2^ = 39.3, df = 2, p < 0.0001) slept inside the house, however the time of entering the house differed. Half of under 5 year olds were in the bed room by 20:30 h, half of 5–18 year olds by 22:00 and half of people >18 year by 23:00 h (Fig. 4). LLINs were used by 100% of under 5 year olds, 85% of 5–18 years and 80% of >18 year olds, which was statistically different (χ^2^ = 29.4, df = 2, p < 0.0001).

### Difference between villages

The movement of people differed significantly between the villages (β = 111.76, se = 12.34, p = 0.0004). In Tuguivili village, more people moved beyond the village on any given 24-hour period compared to Haleta village. This was particularly relevant for overnight trips (as mentioned earlier). For those who remained in the village, there was no statistical difference between the two villages in the percentage of those inside the house (Tuguivili = 15%, Haleta = 19%) and inside the peri-domestic area (Tuguivili = 68%, Haleta = 72%) during 20:00–21:00 h (middle of the evening).

### Quantifying human-vector interactions

**Figure 5:**
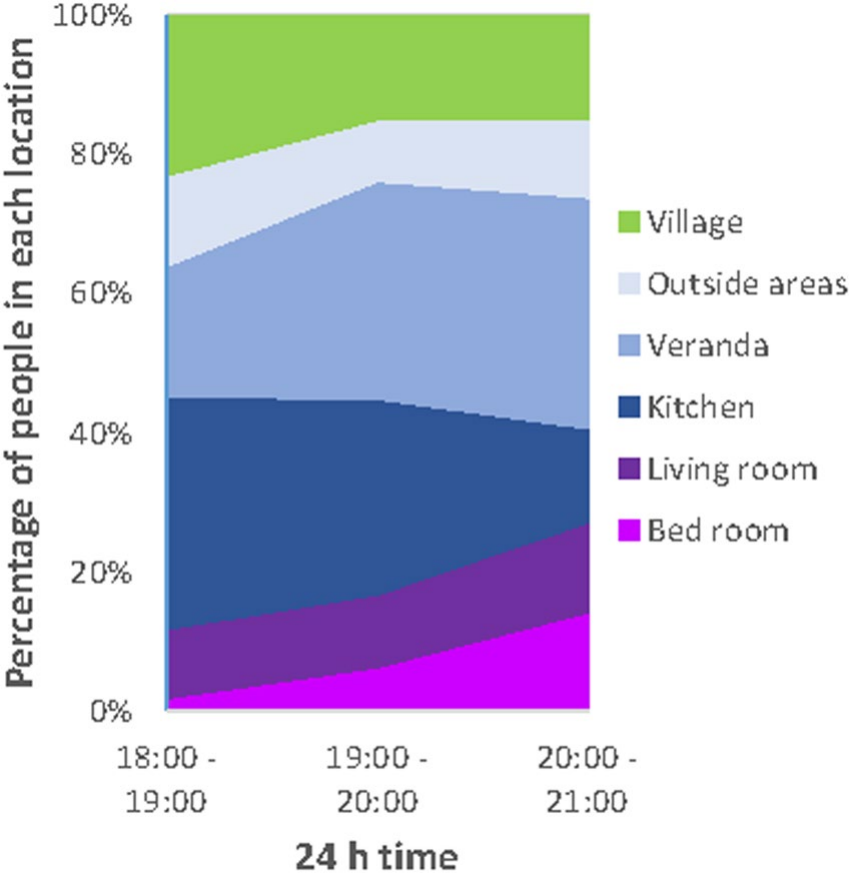
Villager locations during peak mosquito biting timeparticipants inside the house acrosss (1800–2100 h).

Between 18:00–21:00 h, 18% of people were inside the house and 82%were outside (64%in peri-domestic areas, 18% in village) (Fig. 5). Of the 18% indoors only 7% were in the bedroom with access to an LLIN. Previous entomological studies from these villages documented that *An. farauti* is highly outdoor and early biting, with 0.8 bites/hour indoors and 2.1 bites/hour outdoors and 76% of all bites occurring between 18:00 h and 21:00 h^21^. Using this data to quantify human-vector interactions, on any given night 3.2 bites per person occurred inside and 8.2 bites per person occurred outside. Across the entire study population and across the entire night (18:00 to 06:00 h), 16% of all bites occurred inside and 84% outside. The proportion of bites that occurred inside tended to differ by age, with 24% (±18%) of bites for under 5 year olds, 21% (±17%) of bites for 5–18 ys and 12% (±12%) of bites for over 18-year olds occurring inside the house, noting the wide standard errors around these estimates.

## Discussion

This study documented significant movement of people both within and beyond two villages in the Solomon Islands. Although almost everyone in the study had access to and ultimately slept under an LLIN, only 7% of people were under an LLIN during the 18:00–21:00 h peak biting period when 76% of *An. farauti* bites occur^21^. Transmission risk is a product of both the location of people and the biting activity of mosquitoes. By quantifying human-vector interactions in this manner, the greatest exposure to mosquito bites was determined to occur early in the evening when over half of people were on the veranda or in the adjacent external kitchen building, neither of which offer protection from mosquitoes. Thus, a large protection gap exists despite universal access and near universal use of LLINs for most people by virtue of their location within the outdoor peri-domestic area when most malaria mosquitoes are seeking blood meals.

Understanding small-scale heterogeneities in transmission requires high resolution human spatial data^9^. This is the first study to record the daily patterns of where and when people are, within and beyond villages, in the Solomon Islands. In the Solomon Islands, 80% of the population live in rural areas in nuclear families (mean of 5.3 people), 5% of whom are formally employed (by the government or in the private sector^22^). Houses are constructed on stilts with 99% of roofs constructed with iron sheets or leaf-thatch and 78% of walls made of wood or leaf-thatch with 96% of households having at least one LLIN^23^. The study population was representative of the Solomon Island population: 10% of participants were formally employed and resided in households with a mean of 5.4 people per house, 100% of houses were constructed on stilts with roofs of iron or leaf-thatch and with walls made of wood or leaf-thatch. All (100%) of participating households had LLINs. The methodology employed in this study (questionnaires and movement diaries) collected high quality fine-scale data on human movements by time and locations^24^, and focused on specific areas around and within the home in remote village settings. In contrast, mobile phone records and GPS technologies are unlikely to capture human movement with such fine granularity^17^, and further there is limited cell phone coverage in rural Solomon Islands. This study documented both frequent human movement within a village, and travel beyond the village boundaries in which a third of villagers spent an average of 3.6 nights away from the home village over a 2-weeks period. Malaria control programmes therefore need to protect people within villages and when travelling beyond the village, in particular between high and low transmission areas.

This study identified verandas and kitchens in the peri-domestic space as where most people are when malaria vectors are most actively seeking blood meals. These areas of high vector-human contact are open and exposed and are where residual transmission is maintained. These areas should be the focus of additional vector control strategies. Malaria programs can consider extending the usual application of residual insecticides to beyond the inside walls of house to include targeted spraying of the exterior walls of verandas and kitchens. World Health Organisation guidelines recommend that countries determine the locations where vectors bite and rest to determine locations for residual insecticide applications^25^. The findings of this study suggest that IRS should include kitchens and verandas in addition to the inside walls of houses. Alternatively, novel control methods such as insecticide-treated durable wall linings^26^, spatial repellents^27–29^, insecticidal paints or screening to mosquito-proof verandas and kitchens could be evaluated.

This study linked entomological data with fine scale human movement studies to define the locations where residual transmission occurs and to identify the locations where future strategies with the potential for preventing mosquito bites should focus. Gaps when LLINs are not protective were identified during the early evening when most vectors seek blood meals. During this time, most people are outside, in peri-domestic areas (e.g., on verandas or in the kitchen area) near their houses. The size of this protection gap will vary depending on the behaviours of the human and vector populations^10^. While LLINs continue to provide significant protection from malaria, supplemental vector control strategies are needed to accelerate transmission reduction. Even after malaria is eliminated, vector surveillance and control along with human behaviour research needs to be maintained in receptive areas where there is significant risk of importation of parasites by infected people^13,30^.

## Methods

### Study site

**Figure 6:**
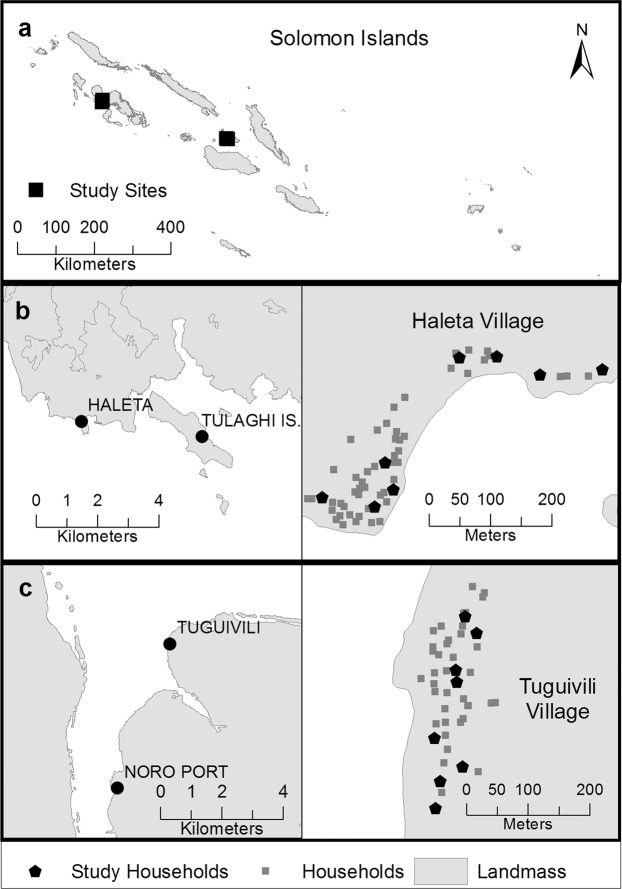
Map of (**a**) the Solomon Islands showing (**b**) location of Haleta village on Nggela Sule Island in Central Province (9°5′56″S, 160°6′56″E) on the right and detailed map of the positions of all households and study housholds on the left and (**c**) location of Tuguivili village on New Georgia Island in Western Province (8°11′49″S, 157°12′54″ E) on the left with the locations of the all households and study households on the right.

The study was conducted in two typical Solomon Island coastal villages (Haleta and Tuguivili; Fig. 6). Haleta village (population 366 people) is located on Ngella Sule Island in Central Province (9°5′56″ S, 160°6′56″ E). Central Province had an Annual Parasite Incidence (API) of 280 cases per 1,000 persons during 2015^31^. The average human biting rate of *An. farauti* was 15 bites per person per night (b/p/n) during 2011–2014^21^ Tuguivili village (population 167 people) is on New Georgia Island in Western Province (8°11′49″ S, 157°12′54″ E). Western Province had an API of 30 cases per 1,000 persons during 2015^31^. The human biting rate of *An. farauti* was 3 b/p/n during 2015–2017^13^. The mean daily coastal temperature for the Solomon Islands ranges between 24 °C and 30 °C with a mean of 27 °C and rainfall between 3000–5000 mm^32^. *Anopheles farauti* is the dominant malaria vector in the study villages (and the Solomon Islands as documented in previous studies^33^).

Greater than 80% of the Solomon Island population lives in rural villages. The villages are generally small, averaging 68 residents in 12.4 households. The economy is largely non-monetary with 96% of households practicing subsistence farming with 60% also fishing^22^. Annual household income in 2013 from selling crops, handicrafts and fish was USD 968 (SBD8011)^34^. More than 98% of Solomon Island residents are Christians and more than 95% are Melanesian^22^. Houses in the Solomon Islands are predominantly constructed on stilts with timber frames and timber or leaf-thatched walls, and with roofs of iron sheet or leaf-thatch. Five percent of rural households have electricity and fewer than 1% have television^35^. The houses have large open eaves and are homogenous in size with 2–3 bedrooms.

### Interviews and movement diaries

The lead author (EJMP) conducted the recruitment and enrolment process after permission was obtained from the village chief to conduct research activities within the village. The village was divided into geographic zones, and households meeting the inclusion criteria were selected from each zone (see Fig. 6). The inclusion criteria included household heads having basic literacy, all household members being permanent residents of the study village, and being willing to provide informed consent. Households meeting the inclusion criteria were selected from each village zone.

A total of 86 people were enrolled from 16 households distributed across both villages (Fig. 6). The location of each resident was recorded for 14 days during July 2017. The demographic information of each household was captured with an initial questionnaire that included: number of household occupants, their age, gender and use of LLINs (number/household).

The location of each resident was recorded by the household heads using daily movement diaries for the 14-day period. All household heads received training in the use of the diaries but were not compensated for recording human movement data. Movement diary entries were recorded as short answers in English, the language of instruction in the Solomon Islands. Prior to the start of the study, data collection and recording were trialled for 2 days under the supervision of the lead author and translator to ensure comprehension of the data recording instructions and to ensure accurate recording of data. During the study, the lead author and the translator lived in the study villages and visited households in the evening to answer questions and to check progress including daily inspections of the movement diaries to ensure complete data capture for all recording periods for all household members. The informant recorded his/her movements and approximately 4 other household residents, using both direct observations and reported recall. The validity of diary entries was confirmed by spot checks of diary entries recorded by the informant against independent observations of residents’ locations by the senior author. The lead author and translator were fluent in *Solomon Pijin*, the lingua-franca used for discussions, clarifications and training.

During the day (06:00–18:00 h), data were recorded in 3 blocks of time, each of 4 h duration. During the evening (18:00–00:00 h), data were recorded hourly. A single block of time was recorded for the night (00:00 to 06:00) when limited people movement occurred. For each time period, the household head observed the location of the household members. Household heads acting as informants were provided synchronised watches set to chime hourly during movement recording periods to remind the household head to complete the diary every hour. The predominant location of each participant was recorded as short open text answers which were subsequently categorised into 4 main broad geographic areas (“Inside House”, “Peri-domestic area”, “Village” and “Beyond Village”, see Table 1). Data entries that could have multiple interpretations were clarified in consultation between informants and study investigators.

### Statistical analysis

The age distribution of participants was described and compared with the national baseline average using a chi-squared contingency table (*chisq. test*). Baseline population data was accessed from projected figures for 2017 based on the 2009 census data^22^.

Generalised linear models (GLMs) with Gaussian distribution were used to analyse differences in: 1. the temporal location of participants compared between villages; 2. the number of nights that participants spend away from their home village by gender and village;3. the age of participants who travelled compared with the age of all participants; 4. the location of all participants across different times of the day and into the evening (06:00–22:00 h); 5. the location of all participants across different hours of the evening (18:00–00:00 h), 6. the location of participants between weekend and weekday days; and 7. the participants locations inside the house between age groups throughout the evening (18:00–00:00 h). The significance of the interaction between location of the participants and time of the day was analysed using a Chi-square test (*anova*) that compared the fit of two nested poisson GLM models. This statistical method was chosen because both the factors of location and time of the day were categorical. The eventual sleeping location and the usage of LLIN compared by age group used a chi-squared contingency table (*chisq. test*).

#### Quantifying human-vector interactions

Prior to conducting the human movement surveys, the biting behaviour of the local *An. farauti* population was quantified and published^21^. The proportion of human contact with mosquito bites occurring indoors (π_i_) was calculated by weighting the mean indoor and outdoor biting rate of *An. farauti* throughout the night by the proportion of humans indoors and outdoors at each time period (indoors being humans inside the houses) and outdoors being humans in the peri-domestic area to match with the mosquito data collected “inside” and “outside” of houses): ${\pi }_{i}=\sum [{I}_{t}{S}_{t}]/\sum [{O}_{t}(1-{S}_{t})]+{I}_{t}{S}_{t}$; where *S* = the proportion of humans indoors, *I* = the total number of mosquitoes caught indoors, *O* = the total number of mosquitoes caught outdoors [see^10^ for more detail].

### Study limitations

This study had several limitations. This was a small study documenting the locations of 86 people from 16 households in two villages across a single 14-day period. Both villages were ‘typical’ rural villages in a ‘typical period of village life’ where people live in family groups on their customary land and engaged in the rural subsistence economy (96% of the 80% rural population of the Solomon Islands practices subsistence agriculture^22^). However, the study may have been improved by monitoring the movements of a greater number of households in villages across more provinces. The single 14-day period study period did not allow data collection from different periods of the year, seasons or during social/cultural events. However, the lack of distinct and/or extended wet and dry seasons in the Solomon Islands coupled with the near universal, practice of subsistence farming results in rural village populations having very regular and predictable daily/weekly activity patterns (e.g, tending gardens, selling excess produce at local markets and/or fishing). Almost everybody returns to their village each night and engages with their extended family who live together in the village. Extending the study to include a period of enhanced social movement, for example at Christmas or another religious or cultural event, when former residents return to their ‘home’ village, would have provided information about people movements in ‘non-typical’ periods.

The movement data was composed of both direct visual observations and self-reported recall. Self-reported recall may introduce social desirability bias where respondents report locations that they think are ‘correct’ for the purpose of the study. For example, self-reported use of mosquito bednets is often over-reported (as they are likely to have been in this study). However, during the evening period when most *An. farauti* bites occur, most people were recorded in directly observable locations. As there is no obvious ‘correct’ answer for locations of individuals, social desirability bias would be minimal. This study did not incorporate cell phone generated data which could have provided information on destinations of village residents moving longer distance. However, as the study focus was on detailing at a very fine scale human locations within villages, cell phones would not have provided detailed information on locations of humans within houses or have distinguished locations within the peri-domestic area. Future research should consider these limitations when designing additional studies.

### Ethical approval and informed consent

Ethics approval for this study was provided by the Solomon Islands Health Research and Ethics Review Board (No. HRE046/16) and the James Cook University Research Ethics Committee (No. H6840). All research was performed in accordance with relevant guidelines and regulations of these research boards, and as stipulated in the approvals. Each participant completed a written informed consent form before participating in the surveys, noting that consent for minors and children was provided by the household head. The village chief gave permission for work in the village and also provided permission for each selected household to be involved.

## Data Availability

The anonymised and categorised datasets supporting the conclusions of this article are available in the JCU Tropical Data Hub repository: 10.25903/5cbe61a46c51f.
